# Integrin–Talin axis regulates mechanosensory presynaptic development and behavioral habituation to mechanical stimulation

**DOI:** 10.1093/g3journal/jkag154

**Published:** 2026-06-16

**Authors:** Jonathan Amezquita, Nelson A Ayala, Muriel Desbois, Neal D Mathew, Karla J Opperman, Brock Grill

**Affiliations:** Norcliffe Foundation Center for Integrative Brain Research, Seattle Children's Research Institute, Seattle, WA 98101, United States; Molecular and Cellular Biology Graduate Program, University of Washington, Seattle, WA 98101, United States; Norcliffe Foundation Center for Integrative Brain Research, Seattle Children's Research Institute, Seattle, WA 98101, United States; Department of Pharmacology, University of Washington School of Medicine, Seattle, WA 98101, United States; School of Life Sciences, Keele University, Keele, Stafforshire ST5 5BG, United Kingdom; Norcliffe Foundation Center for Integrative Brain Research, Seattle Children's Research Institute, Seattle, WA 98101, United States; Department of Pediatrics, University of Washington School of Medicine, Seattle, WA 98101, United States; Norcliffe Foundation Center for Integrative Brain Research, Seattle Children's Research Institute, Seattle, WA 98101, United States; Norcliffe Foundation Center for Integrative Brain Research, Seattle Children's Research Institute, Seattle, WA 98101, United States; Department of Pharmacology, University of Washington School of Medicine, Seattle, WA 98101, United States; Department of Pediatrics, University of Washington School of Medicine, Seattle, WA 98101, United States

**Keywords:** adhesome, RPM-1, MYCBP2, Talin, Kindlin, integrin, mechanosensory neuron, synaptic branch, synapse formation, habituation, learning, *C. elegans*

## Abstract

From invertebrates to mammals, integrin signaling plays important roles in neuron development. β-Integrins are regulated by binding extracellular matrix and through intracellular activation or inside-out signaling via Talin and Kindlin. While Integrins are known to regulate synapse formation and learning, much less is known about effects of Integrins, Talin and Kindlin on presynaptic branch formation and outcomes on behavior. Here, we use *Caenorhabditis elegans* as an *in vivo* model to study how the PAT-3/UNC-112/TLN-1 (β-integrin/Kindlin/Talin) signaling axis affects formation of presynaptic branches. Using a CRISPR-based, cell-specific degradation approach, we demonstrate that this Talin axis functions cell-autonomously in mechanosensory neurons to regulate presynaptic branch formation. Transgenic findings indicate TLN-1 inside-out signaling also influences branch formation. Thus, both impairing and activating Talin result in branch defects suggesting that balanced TLN-1 axis signaling is required for branch development. Genetic interaction and developmental time-course studies revealed that the TLN-1 axis functions in parallel with the RPM-1 (MYCBP2) ubiquitin ligase signaling hub to regulate both branch and presynaptic bouton formation. Consistent with the TLN-1 axis affecting presynaptic development in mechanosensory neurons, TLN-1 influences behavioral habituation to repeated low-threshold mechanical stimulation. Collectively, our findings indicate that an Integrin–Talin axis functions cell-autonomously in mechanosensory neurons to regulate presynaptic development and behavioral habituation.

## Introduction

β-Integrins are transmembrane receptors that bridge the extracellular matrix (ECM) and intracellular environments, mediating complex interactions essential for cellular organization and function. Integrins display bidirectional signaling: (1) Signaling promoted by ECM also called outside-in signaling; and (2) activation of Integrins by intracellular signaling through Talin and Kindlin also referred to as inside-out signaling (Calderwood et al. 1999; Rogalski et al. 2000; Cram et al. 2003; Montanez et al. 2008; Calderwood et al. 2013). The Integrin/Kindlin/Talin signaling axis regulates several processes including adhesion, migration, cell proliferation, and survival (Hynes 2002; Kim et al. 2003; Arnaout et al. 2005). Integrins are implicated in several diseases including cancer, inflammatory disorders, and fibrosis. Importantly, growing evidence links integrin signaling dysfunction to neurodevelopmental disorders and neurodegenerative diseases (Clegg et al. 2003; Jaudon et al. 2021; Amezquita et al. 2024; Cui et al. 2024).

Within neurons, Integrin signaling plays prominent roles in axon guidance and outgrowth, as well as synapse formation and plasticity (Park and Goda 2016; Lilja and Ivaska 2018). Inactivation of Integrin β1 and β3 result in impaired excitatory synaptic density and maintenance, long-term potentiation, and impaired learning behaviors in rodents (Chavis and Westbrook 2001; Huang et al. 2006; Warren et al. 2012). In contrast to Integrins, much less is known about the roles of Talin and Kindlin in neurons. Inactivation of Talin in chick dorsal root ganglion (DRG) neurons leads to reduced filopodial motility in growth cones (Sydor et al. 1996) and in cultured rat DRG neurons overexpression of Talin or Kindlin enhances neurite growth (Tan et al. 2012; Tan et al. 2015). Talin also plays a presynaptic role regulating actin dynamics and synaptic vesicle endocytosis (Morgan et al. 2004). Interestingly, studies utilizing both Drosophila and mice have demonstrated that presynaptic homeostatic plasticity is driven by Integrin–Talin signaling (Orr et al. 2022). At present, whether Talin and Kindlin regulate presynaptic branch formation during development remains unknown. Finally, while β-Integrins have been shown to affect learning in rodents, it remains unclear if Talin or Kindlin influence behavioral habituation or other forms of learning in response to mechanical stimulation.

Individual components in the Integrin/Kindlin/Talin axis have been found to regulate axon and synapse development from *Caenorhabditis elegans* through mammals (Chavis and Westbrook 2001; Poinat et al. 2002; Pasterkamp et al. 2003; Cingolani and Goda 2008; Kerstein et al. 2017; Eysert et al. 2021). In *C. elegans*, there are single orthologs of β-Integrin, Kindlin, and Talin referred to as PAT-3, UNC-112, and TLN-1, respectively (Fig. 1a). Here, we use CRISPR-based, cell-specific degradation to demonstrate that the entire PAT-3/UNC-112/TLN-1 axis functions cell-autonomously in mechanosensory neurons to regulate presynaptic branch formation. Our findings further indicate that activation of TLN-1 (*i.e.* inside-out signaling) affects branch formation. Genetic interaction and developmental time-course studies demonstrate that TLN-1 functions in parallel with a key player in presynaptic development, the RPM-1 ubiquitin ligase signaling hub. Finally, our results indicate that TLN-1 effects on presynaptic formation result in defective behavioral habituation to low threshold mechanical stimulation.

**Fig. 1.**
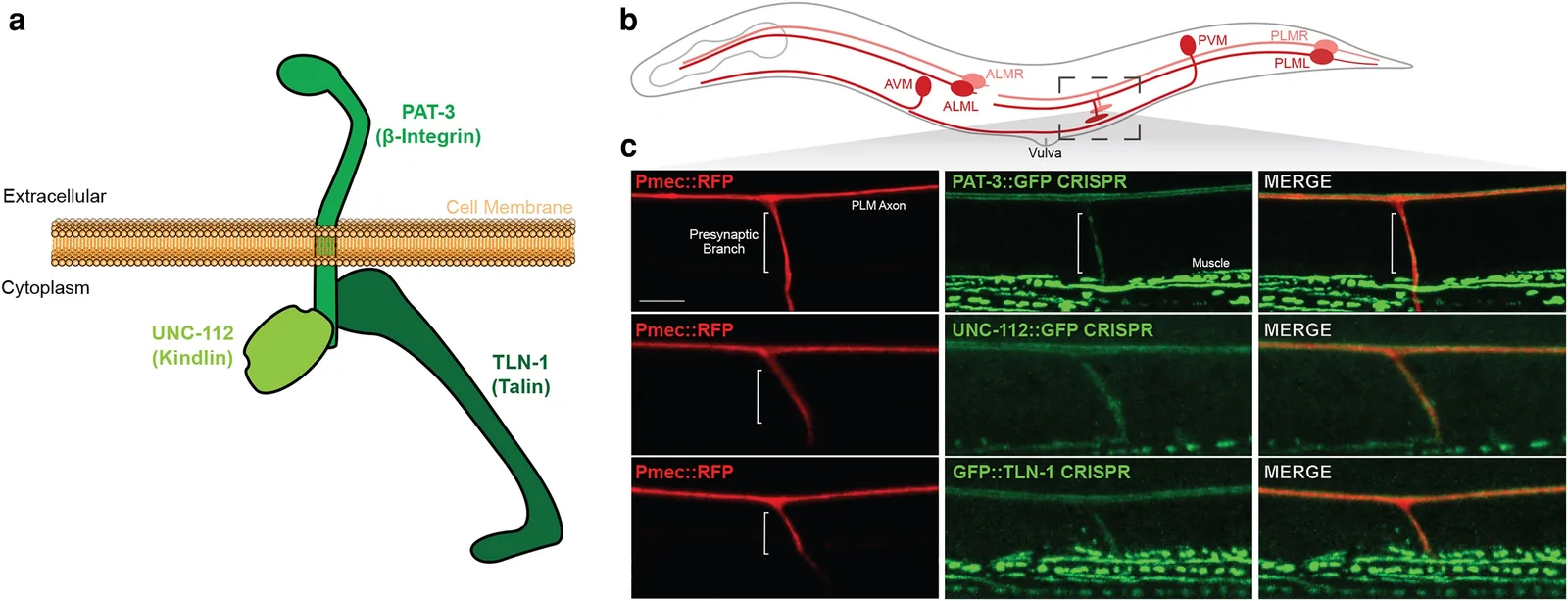
PAT-3/UNC-112/TLN-1 axis components are localized to presynaptic branches of PLM mechanosensory neurons in *C. elegans*. a) Illustration of PAT-3/UNC-112/TLN-1 axis showing PAT-3 (β-Integrin, green), UNC-112 (Kindlin, light green), and TLN-1 (Talin, dark green) which were CRISPR engineered with GFP. Adapted from Amezquita et al. (2024). b) Schematic of *C. elegans* mechanosensory neurons highlighting collateral presynaptic branches of PLM neurons visualized using P*mec-7*::mRFP (*jsIs973*) (dashed box). c) Representative images showing PAT-3::GFP, UNC-112::GFP, and GFP::TLN-1 (middle panels) localized to collateral presynaptic branches (bracket) of PLM neurons in young adults. Images show only a single PLM branch on 1 side of the animal. Note PAT-3/UNC-112/TLN-1 axis components also show prominent expression in the primary PLM axons and body wall muscles. Scale bar 5 μm.

## Methods

### Genetics and strains

C. *elegans*  N2 isolate was used for all experiments. Animals were maintained using standard procedures. The following mutant alleles were used: *rpm-1(ju44)* V and *tln-1(ok1648)* I. The following integrated transgene was used: *jsIs973* [P_mec-7_mRFP] III. The following MosSCI transgene was used: *itSi953* [P_mec-18_vhhGFP4::ZIF-1::operon-linker::mKate::tbb-2 3′UTR+Cbr-unc-119(+)] II, which we refer to as *mecDEG*. All MosSCI transgenes were inserted into *ttTi5605* on LG II. The following clustered regularly interspaced short palindromic repeats (CRISPR)/Cas9 alleles were used: *unc-112(bgg68* [UNC-112::GFP CRISPR]), *pat-3(bgg86* [PAT-3::GFP CRISPR]), and *tln-1(zh117* [GFP::TLN-1 CRISPR]).

All transgenes and CRISPR alleles used for specific experiments are described in Supplementary Table 1. All mutants and transgenic lines were outcrossed 4 or more times prior to experiments. Animals were grown at 23 °C for genetic analysis. Sequences of all genotyping primers for mutants and CRISPR strains can be found in Supplementary Table 2.

### Molecular biology and transgenics

Five fragments of genomic *tln-1* DNA (gDNA) (pBG-454 to pBG-458) were amplified from N2 genomic DNA using iProof High-Fidelity DNA polymerase (BioRad) or Q5 High-Fidelity DNA Polymerase (NEB). Agilent point mutagenesis kit was used to introduce T1767E (pBG-459) and E1770A (pBG-460) point mutations into the relevant *tln-1* gDNA fragment. Wildtype or point mutated *tln-1* fragments were assembled to generate full length *tln-1* gDNA clones in a pCR8 vector using HiFi DNA assembly (NEB) (pBG-GY1088, pBG-GY1096, pBG-GY1097). Using gateway technology (LR clonase II, Invitrogen), *tln-1* gDNA constructs were recombined with a plasmid containing P_rgef-1_FLAG and unc-54 3′UTR (pBG-GY134) to generate the following plasmids: (1) P_rgef-1_FLAG::TLN-1 (pBG-GY1100), (2) P_rgef-1_FLAG::TLN-1 T1767E (pBG-GY1101), and (3) P_rgef-1_FLAG::TLN-1 E1770A (pBG-GY1102). All constructs were fully sequenced prior to generation of transgenic strains.

Transgenic extrachromosomal arrays were generated using standard microinjection procedures for *C. elegans*. Injection conditions and transgenes construction details are specified in Supplementary Table 3.

### CRISPR/Cas9 engineering

CRISPR alleles were generated using the *dpy-10* co-CRISPR technique and direct injection of Cas9 ribonucleoprotein complexes. The injection mixtures included tracrRNA (IDT), a repair template (either PCR product or ssODN from IDT Ultramers), and recombinant Cas9 protein purified from Rosetta 2 *E. coli* (Millipore EMD, #71397). Prior to injection, ribonucleoprotein complexes were incubated at 37 °C for 15 min. Gene modifications were validated by sequencing, and edited strains were backcrossed at least 4 times with N2 animals. Details of CRISPR injection conditions can be found in Supplementary Table 3, and sequences for all crRNAs and repair templates are listed in Supplementary Table 4.

### Airyscan super-resolution imaging with GFP tagged PAT-3/UNC-112/TLN-1

Young adult PAT-3::GFP, UNC-112::GFP, or GFP::TLN-1 CRISPR engineered animals carrying the transgene *jsIs973* [P_mec-7_mRFP] were anesthetized using 10 μM levamisole. Animals were mounted on 3% agarose pads on glass slides with coverslips. Super-resolution images were captured using a Zeiss LSM 900 microscope with Airyscan 2 super-resolution under a 63× oil-immersion objective.

### Analysis of PLM presynaptic branch formation

The transgenic strain *jsIs973* [P_mec-7_mRFP] was used to label PLM neurons for analysis of presynaptic branches in young adult animals. Branch defects were scored by examining whether the PLM branch reached the ventral nerve cord. Partial or absent branches were scored as branch defects.

Branch defects were scored using epifluorescent microscopy with young adult animals anesthetized with 10 μM levamisole in M9 buffer. Animals were mounted onto 2% agar pads on glass slides with coverslips. Animals were visualized using a Leica DM5000 B (CTR5000) microscope equipped with a 40× oil-immersion objective. 5–6 counts of 20–30 PLM neurons were scored for each genotype over 3 independent biological experiments.

For image acquisition of branch defects, young adult animals were anesthetized using either 10 μM levamisole or a 3% (v/v) solution of 1-phenoxy-2-propanol in M9 buffer. Animals were mounted on 3% agarose pads on glass slides with coverslips. Images were captured using a Zeiss LSM 710 equipped with 40× oil-immersion objective.

### Quantitation of GFP::TLN-1 levels in mechanosensory neurons using Airyscan super-resolution imaging

Young adult animals expressing CRISPR engineered GFP::TLN-1 with or without *mecDEG* were anesthetized with 10 µM of levamisole in M9 buffer and mounted on 5% agarose pads on glass slides with coverslips. PLM soma and proximal axon were imaged using a Zeiss LSM 900 equipped with a 40× oil-immersion objective and Airyscan 2 super-resolution imaging. Images were acquired and processed using Zeiss Zen software with identical acquisition and processing settings across genotypes. Zeiss Zen was used to generate color-coded intensity plots of GFP::TLN-1.

### Developmental time-course analysis

*jsIs973* [P_mec-7_mRFP] labeled PLM neurons, and we examined both presynaptic branch and presynaptic bouton morphology during development. Developmental time-course studies were performed as previously described (Borgen et al. 2019). Animals were synchronized by collecting freshly hatched L1 larvae every 10–15 min and culturing on plates at 23 °C to desired time points. At 6, 16, and 48 h post-hatch (PH), animals were mounted in 5 μM levamisole (6, 16 h PH) or 10 μM levamisole (48 h PH) in M9 buffer on 3% agarose pads.

To quantify presynaptic bouton defects, we examined whether presynaptic branches reached the ventral nerve cord and then specifically scored whether boutons were formed. Branches with presynaptic boutons/varicosities were considered wild type. In cases where no bouton was distinguishable, the presynaptic terminal was recorded as destabilized even if the branch was present. Three independent counts of 15–20 PLM neurons were scored at each time point for all genotypes. Scoring was performed using a Leica DM5000 B (CTR5000) epifluorescent microscope equipped with a 40× and 63× oil-immersion objective.

Representative images for each genotype at select developmental time points were acquired with a Zeiss LSM 900 microscope under a 63× oil-immersion objective. Z-stack images were collected, and maximum intensity projections are shown. Image analysis was done using ZEISS Zen Microscopy Software (RRID:SCR_013672).

### Habituation

Mechanosensory habituation experiments were performed using MWT with a plate tapping system as described previously with minor modifications (Giles et al. 2015). For all genotypes, a total of 10 experiments were performed across 5 independent days of data acquisition. For a single experiment, age-synchronized animals (∼50–100) were cultivated at 23 °C from egg until gravid adult (72–75 h post-hatch) on 6 cm NGM plates containing 50 μL of E. coli (OP50).

Computational behavioral recording was performed using MWT for a total time of 550 s on NGM culture plates without food. After an initial 100 s of baseline recording, MWT was used to apply 45 tap stimuli given with a 10 s inter-stimulus interval. Response to tap was measured as the fraction of animals that reversed their locomotion within 2 s of a plate tap (reversal probability). Initial tap response was defined as the reversal probability of animals during the first tap. For each plate, analysis of the tap response was done using Choreography (MWT analysis software) with custom written scripts (Octave). Habituation was assessed by the overall reversal probability response which is represented by the exponential best-fit curve and the final habituation level, defined as the asymptote of the fit at the final stimulus.

All strains analyzed, apart from wild-type, contained the *jsIs973* transgene which was used to monitor/confirm synaptic phenotypes prior to testing habituation.

### Statistical analysis

All statistical analysis was performed using GraphPad Prism software.

#### Branch defects

For analysis of PLM branch defects (%) in young adult animals, a minimum of 5 counts of 20–30 PLM neurons were scored for each genotype over a minimum of 3 independent biological experiments. Histograms represent the mean, dots represent individual counts, and error bars represent the SEM. Statistical comparisons were performed using two-way ANOVA with Bonferroni correction for multiple comparisons where needed. Significance was defined as *P* < 0.05.

#### GFP::TLN-1 and Pmec::RFP fluorescence intensity measurements in PLM neurons

ROIs were manually drawn in Zeiss Zen Software (v3.9.2) using the spline tool around PLM soma and creating 4.5 µm^2^ box around proximal axon using maximum intensity projections. Reported mean fluorescence measurements were quantified in soma and axon ROIs. Images for 10–12 PLM neurons were analyzed for each genotype and acquired over 3 independent biological experiments. Statistical analysis was performed using unpaired, two-tailed Student's *t*-test. Significance was defined as *P* < 0.05.

#### Developmental time course

For developmental time-course analysis, we evaluated the percentage of PLM neurons lacking presynaptic boutons in all genotypes tested at 6, 16 and 48 h PH time points. Histograms represent averages from 50–60 PLM neurons from 3 or more independent imaging session for each genotype. Statistical comparisons were performed using Fisher's exact test. Significance was defined as *P* < 0.05. Fisher's exact test was chosen because developmental time course studies showed presence or absence of presynaptic phenotypes. We also chose this test because the technical difficulty and throughput of these experiments did not allow us to conduct enough independent experiments to apply other statistical tests such as a two-way ANOVA.

#### Habituation

Habituation profiles, initial tap response, reversal probabilities and final habituation level (asymptote) were recorded for 10 plates (∼50–100 animals/plate) for all genotypes (*n* = 10). Data was collected from 5 independent biological experiments performed on different days (2 plates per genotype per day). Dots represent mean reversal probability at individual time points, and error bars represent SEM for each genotype. Statistical comparisons for habituation profiles, initial tap response, and final habituation levels were performed using two-way ANOVA with Bonferroni correction for multiple comparisons. Significance was defined as *P* < 0.05. Individual tracking events that were not acquired properly by MWT because the tapper failed to fire or animal tracking was momentarily lost were excluded.

## Results

### PAT-3/UNC-112/TLN-1 signaling axis localizes to presynaptic branches of PLM mechanosensory neurons

We previously used CRISPR engineering to generate GFP-tagged PAT-3, UNC-112, and TLN-1, which were used to examine their respective expression and localization in *C. elegans* mechanosensory neurons (Walser et al. 2017; Amezquita et al. 2024). Consistent with prior studies, we observed PAT-3::GFP, UNC-112::GFP, and GFP::TLN-1 in the primary axons of PLM mechanosensory neurons (Fig. 1b) (Amezquita et al. 2024). Here, we used Airyscan super-resolution imaging with live *C. elegans* to determine whether these players are localized to subcellular compartments relevant to presynaptic branch formation in mechanosensory neurons.

C. *elegans* have 2 PLM neurons in the tail ganglia that extend primary axons anteriorly along the left and right sides of the animal (Fig. 1b). Each axon gives rise to a collateral branch that forms presynaptic terminals. We visualized PLM axon morphology with a P*mec-7*::mRFP (*jsIs973*) transgene. Using Airyscan super-resolution imaging, we detected components of the PAT-3/UNC-112/TLN-1 axis in the presynaptic branch (Fig. 1c, Supplementary Fig. 1). We were unable to further assess whether these components are expressed at presynaptic boutons due to prominent expression of all components in muscles (Fig. 1c, Supplementary Fig. 1). Nonetheless, our results using CRISPR engineering and super-resolution microscopy indicate that the PAT-3/UNC-112/TLN-1 axis is localized to the PLM presynaptic branch, an observation that is consistent with a potential role in presynaptic branch development.

### CRISPR-based cell-specific degradation of PAT-3/UNC-112/TLN-1 axis results in presynaptic branch defects in mechanosensory neurons

Next, we evaluated whether impairing the PAT-3/UNC-112/TLN-1 axis regulates formation of PLM presynaptic branches. Null mutants for *pat-3* and *unc-112* were previously shown to be lethal, and *tln-1* null mutants have failed to be identified presumably also due to lethality associated with muscle development (Williams and Waterston 1994; Amezquita et al. 2024). As a result, we previously deployed a CRISPR-based, cell-specific strategy for impairing individual members of the PAT-3/UNC-112/TLN-1 axis (Fig. 2a) (Wang et al. 2017; Sepers et al. 2022; Amezquita et al. 2024). Using a mechanosensory neuron-specific promoter, we transgenically expressed an anti-GFP protein degradation module (*mecDEG*) to selectively target and degrade CRISPR-engineered PAT-3::GFP, UNC-112::GFP, and GFP::TLN-1. The *mecDEG* system consists of the SOCS-box adaptor protein ZIF-1 fused with an anti-GFP nanobody. Anti-GFP::ZIF-1 acts as a ubiquitin ligase adaptor that binds to GFP-tagged proteins and targets them for proteasome-mediated degradation by the endogenous Rbx1/Cul2 E3 ubiquitin ligase complex (Fig. 2a)(Wang et al. 2017; Amezquita et al. 2024). This approach allowed us to investigate the role of the PAT-3/UNC-112/TLN-1 axis in presynaptic branch formation. In young adults co-expressing the *mecDEG* system with PAT-3::GFP, UNC-112::GFP, or GFP::TLN-1 CRISPR, we observed presynaptic branch defects in which the branch was either partially or completely absent (Fig. 2b and c). Quantitation indicated that branch defects were significant with cell-specific degradation of each component in mechanosensory neurons (Fig. 2d). In contrast, we did not observe branch defects with several negative controls including CRISPR-engineered GFP fusions on PAT-3, UNC-112, and TLN-1 (PAT-3::GFP, UNC-112::GFP, and GFP::TLN-1), the *mecDEG* system alone, and wildtype (Fig. 2d). Moderate frequency of defects occurred with the degradation of either PAT-3::GFP or UNC-112::GFP. Interestingly, degradation of GFP::TLN-1 resulted in a particularly high incidence of presynaptic branch defects (Fig. 2d). While *C. elegans* contains 7 TLN-1 isoforms, our CRISPR-based degradation approach only targets the full-length *tln-1* a isoform and a truncated *tln-1* b isoform because of the N-terminal GFP tag (Walser et al. 2017; Amezquita et al. 2024). Thus, our findings suggest that impairing one or both of these TLN-1 isoforms leads to branch defects. Importantly, the higher frequency of defects observed when TLN-1 is impaired suggests that TLN-1 could have a particularly important role in presynaptic branch development. Because our *mecDEG* targeting system is expressed specifically in mechanosensory neurons, our results indicate that the PAT-3/UNC-112/TLN-1 axis, which we will refer to as the TLN-1 axis, regulates presynaptic branch formation by functioning cell-autonomously in mechanosensory neurons.

**Fig. 2.**
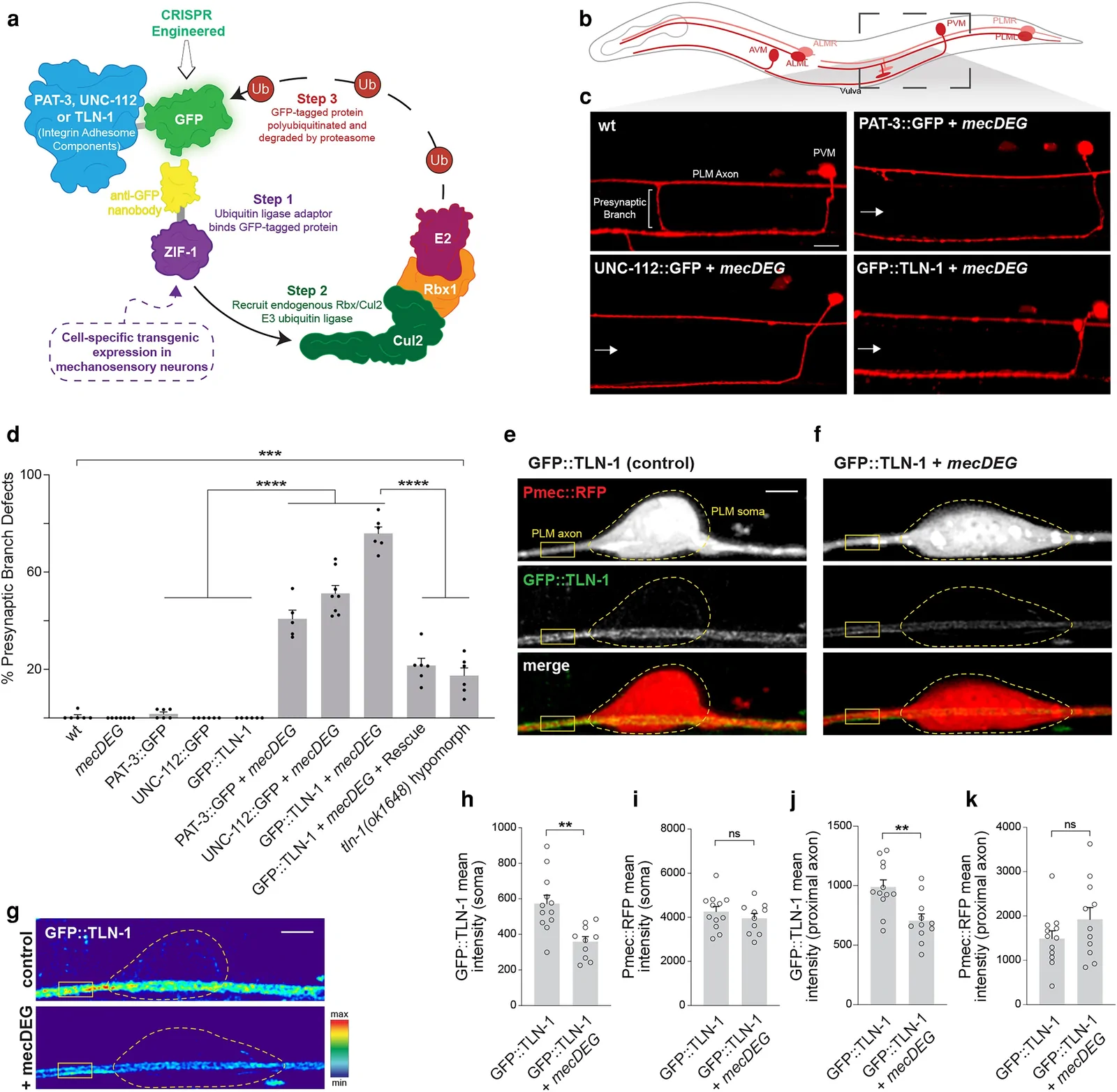
Cell-specific degradation of components in PAT-3/UNC-112/TLN-1 axis results in presynaptic branch defects in mechanosensory neurons. a) Illustration of CRISPR-based, cell-specific protein degradation system, *mecDEG*, used to impair components of PAT-3/UNC-112/TLN-1 axis in *C. elegans* mechanosensory neurons. Adapted from Amezquita et al 2024. b) Schematic highlighting PLM collateral presynaptic branch (dashed box) visualized using P*mec-7*::mRFP (*jsIs973*). c) Representative images of young adults showing presynaptic branch defects (arrows) when *mecDEG* impairs PAT-3::GFP (e.g. PAT-3::GFP+*mecDEG*), UNC-112::GFP, or GFP::TLN-1 CRISPR. d) Quantitation of presynaptic branch defects in PLM neurons for indicated genotypes. Note branch defects occur with *mecDEG* targeting of PAT-3, UNC-112 or TLN-1, and in *tln-1(ok1648)* hypomorphic mutants. e and f) Representative Airyscan super-resolution images of young adults showing GFP::TLN-1 (middle panel) is reduced in PLM neurons where *mecDEG* is expressed. Regions of interest (ROI) used for quantitative analysis (h to k) are shown for PLM soma (yellow, dashed oval) and proximal axon (yellow box). g) Images showing GFP::TLN-1 intensity as super-resolution heatmap (right bar) for indicated genotypes. h–k) Quantitation of GFP::TLN-1 fluorescence intensity for PLM soma (h) and proximal axon (j). P*mec-7*::mRFP fluorescence that fills PLM soma and axon is quantitated as negative control (i and k). For d), means (bars) are shown for 5 or more counts (black dots) with 20 or more animals/count acquired from 3 independent experiments for each genotype. Error bars are SEM. Significance assessed using two-way ANOVA with Bonferroni correction for multiple comparisons. For h to k), shown are means (bars), animals imaged (dots) from 3 independent experiments (10–12 animals/genotype), and error bars for SEM. Significance assessed using Student's *t*-test. ****P < 0.0001, ***P < 0.001, **P < 0.01, ns = not significant. For c, scale bar 5 μm. For e–g, scale bars 2 μm.

While our experiments with *mecDEG* inhibition of the TLN-1 axis relied upon numerous genetic controls, we wanted to further evaluate *mecDEG* effects on protein levels in mechanosensory neurons. To test this, we used Airyscan super-resolution imaging to examine GFP::TLN-1 protein levels in PLM neurons. In young adults where the *mecDEG* was co-expressed with CRISPR engineered GFP::TLN-1, we observed reduced levels of GFP::TLN-1 fluorescence in both the soma and proximal axon compared to GFP::TLN-1 alone controls (Fig. 2e and f; Supplementary Fig. 2). We further performed quantitative super-resolution imaging on GFP::TLN-1 using the soma and proximal axon as regions of interest (ROI). Our quantitative analysis revealed a significant decrease in GFP::TLN-1 signal in both soma (Fig. 2g and h) and proximal axon (Fig. 2g and j) when *mecDEG* was co-expressed with GFP::TLN-1 compared to GFP::TLN-1 controls. In contrast, we did not observe a decrease in control P*mec7*::RFP levels in PLM soma and axon between genotypes (Fig. 2g, i, and k). These findings demonstrate that the *mecDEG* system significantly reduces the levels of CRISPR engineered endogenous GFP::TLN-1 in PLM neurons.

To further validate the branch formation phenotype caused by impairing TLN-1, we took two approaches. First, we performed transgenic rescue experiments using a pan-neuronal promoter to express TLN-1 in animals carrying both the *mecDEG* system and GFP::TLN-1 CRISPR. We chose a pan-neuronal driver for rescue experiments because the *mecDEG* (*Pmec-18*::vhhGFP4::ZIF-1) and P*mec-7*::mRFP used to visualize PLM neuron morphology are driven by mechanosensory neuron specific promoters. By using a pan-neuronal driver, we avoided potential complications that could occur due to the use of too many mechanosensory specific promoters. Presynaptic branch defects caused by using the *mecDEG* to degrade GFP::TLN-1 were rescued by transgenic expression of TLN-1. Because transgenic TLN-1 lacked GFP, it was not targeted by *mecDEG* and allowed evaluation of rescue. Quantitation indicated that transgenic TLN-1 significantly reduced the frequency of branch defects caused by impairing TLN-1 (Fig. 2d). As a second validation strategy, we tested a *tln-1* deletion mutant, *ok1648*, which targets a, c, d, and e isoforms. Prior results indicate that this is a hypomorphic allele for *tln-1* (Amezquita et al. 2024). Significant branch defects were observed in *tln-1(ok1648)* mutants compared to wild-type animals (Fig. 2d). However, the frequency of defects in *tln-1(ok1648)* mutants was lower than with *mecDEG* targeting of GFP::TLN-1 CRISPR, consistent with *tln-1(ok1648)* being a hypomorphic allele.

Taken as a whole, our results support several conclusions. (1) Impairing any component in the PAT-3/UNC-112/TLN-1 axis results in presynaptic branch defects. (2) The *mecDEG* system reduced endogenous GFP::TLN-1 levels as evidenced by quantitative Airyscan super-resolution microscopy. Thus, genetic conclusions on *mecDEG* targeting of the PAT-3/UNC-112/TLN-1 axis are supported by a broad array of genetic controls and quantitative observations on proteins levels in mechanosensory neurons. (3) The *mecDEG* cell-specific strategy indicates the PAT-3/UNC-112/TLN-1 axis functions cell-autonomously in mechanosensory neurons to regulate branch formation. (4) Our results suggest TLN-1 could be a particularly prominent player in presynaptic branch formation.

### Activation of Talin intracellular signaling affects presynaptic branch development

β-Integrins are transmembrane receptors that engage in bidirectional signaling to coordinate a wide range of cellular behaviors and processes. In their inactive state, Integrins adopt a closed conformation in both their extracellular domains and intracellular tails. Activation involves conformational changes triggered by ECM binding, which we refer to as “outside-in” signaling for ease of discussion. In contrast, when intracellular players like Talin are activated “inside-out” signaling occurs with the β-Integrin intracellular domain mediating conformational changes that enhance Integrin–ECM interactions (Kim et al. 2003; Arnaout et al. 2005). Talin exists in a closed conformation where its N-terminal head domain forms autoinhibitory interfaces with its C-terminal rod domain (Fig. 3a) (Lu et al. 2022). Conversely, open-state Talin is recruited to the membrane where it acts as the primary activator for Integrin inside-out signaling (Fig. 3a) (Nayal et al. 2004; Klapholz and Brown 2017). Our results with the *mecDEG* system indicate that TLN-1 functions cell-autonomously in mechanosensory neurons (Fig. 2). As a result, TLN-1 loss of function caused by the *mecDEG* could lead to loss of outside-in signaling, inside-out signaling or both. Here, we test the role of TLN-1 inside-out signaling further.

**Fig. 3.**
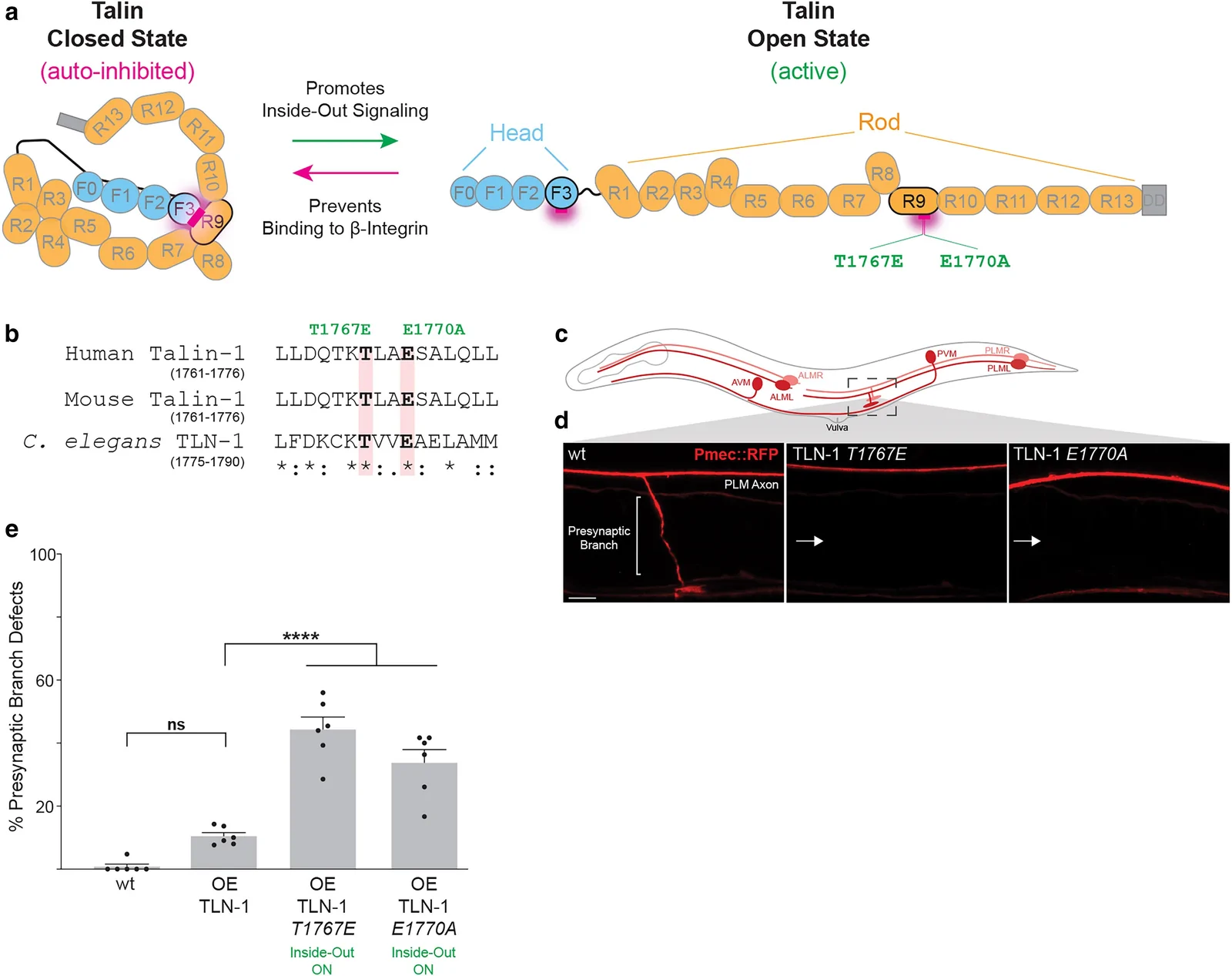
Activating TLN-1 inside-out signaling results in presynaptic branch defects in mechanosensory neurons. a) Schematic of Talin showing N-terminal FERM domain (Head, F0-F3), 13 flexible helix bundles (Rod, R1-R13), and C-terminal dimerization domain (DD). Inactive Talin is auto-inhibited by F3-R9 interaction (pink box), which prevents F3 domain from binding β-Integrin. Talin-1 *T1767E* and *E1770A* point mutations prevent auto-inhibition leading to constitutively active Talin that promotes inside-out signaling. b) Sequence alignment of human, mouse, and *C. elegans* Talin/TLN-1 shows conservation of *T1767* and *E1770* in R9 region responsible for auto-inhibition. c) Schematic highlighting the collateral presynaptic branch of PLM mechanosensory neurons (dashed box). P*mec-7*::mRFP (*jsIs973*) labels mechanosensory neurons. d) Representative images of young adults showing PLM presynaptic branch defects for indicated genotypes. Pan-neuronal promoter (P*rgef-1*) was used to transgenically express TLN-1 or constitutively active TLN-1 *T1767E* or TLN-1 *E1770A* point mutants. Note presynaptic branch defects (arrows) occur with overexpression of constitutively active TLN-1. e) Quantitation of presynaptic branch defects in PLM neurons for indicated genotypes. Data collected and averaged from 2–3 transgenic lines for each genotype. Mean (bars) are shown for 5 or more counts (black dots) with 20 or more animals/count for each genotype. Error bars indicate SEM. Significance assessed using two-way ANOVA with Bonferroni correction. ****P < 0.0001, ns = not significant. Scale bar 5 μm.

Previous studies within and outside the nervous system have identified multiple point mutations that prevent Talin auto-inhibition and stimulate inside-out signaling (Goult et al. 2009; Tan et al. 2015; Haage et al. 2018). Sequence alignment of human and mouse Talin with *C. elegans* TLN-1 demonstrated that human Talin T1767 (*C. elegans* T1781) and E1770 (*C. elegans* E1784) are identically conserved residues across species (Fig. 3b). We generated transgenic TLN-1a overexpression constructs engineered with *T1767E* or *E1770A* point mutations, which promote TLN-1 activation. TLN-1 *T1767E* or *E1770A* were overexpressed using the *rgef-1* pan-neuronal promoter. Both TLN-1 *T1767E* and *E1770A* resulted in presynaptic branch defects that were not observed in wild-type animals (Fig. 3c and d). Quantitation demonstrated that branch defects caused by *T1767E* and *E1770A* occurred at significant frequency compared to overexpression of TLN-1 that was not point mutated to activate inside-out signaling (Fig. 3e). Notably, overexpression of TLN-1 trended toward an increase in frequency of branch defects but this did not reach significance compared to wild-type animals (Fig. 3e). These findings demonstrate that activating TLN-1 and promoting inside-out signaling results in impaired presynaptic branch formation. Given our observation that impairing TLN-1 function also results in presynaptic branch defects, our findings suggest that the appropriate balance of TLN-1 signaling is required for presynaptic branch formation.

### PAT-3/UNC-112/TLN-1 axis functions in parallel with RPM-1 ubiquitin ligase signaling hub to regulate presynaptic branch and bouton formation

To better understand how the PAT-3/UNC-112/TLN-1 axis regulates presynaptic branch formation, we next examined genetic interactions between this axis and the RPM-1 ubiquitin ligase signaling hub. RPM-1, like the TLN-1 axis, functions in mechanosensory neurons to regulate presynaptic branch formation. Prior studies demonstrated that presynaptic branch defects occur in *rpm-1* loss-of-function (lf) mutants due to failed synapse maintenance during development (Fig. 4a and b) (Schaefer et al. 2000; Borgen et al. 2019). RPM-1 is orthologous to human MYCBP2 (PAM), which is associated with a recently described neurodevelopmental disorder (AlAbdi et al. 2023). Because impairing the TLN-1 axis results in presynaptic branch defects similar to loss of RPM-1, we tested the functional relationships between RPM-1 and this axis in the context of presynaptic branch formation.

**Fig. 4.**
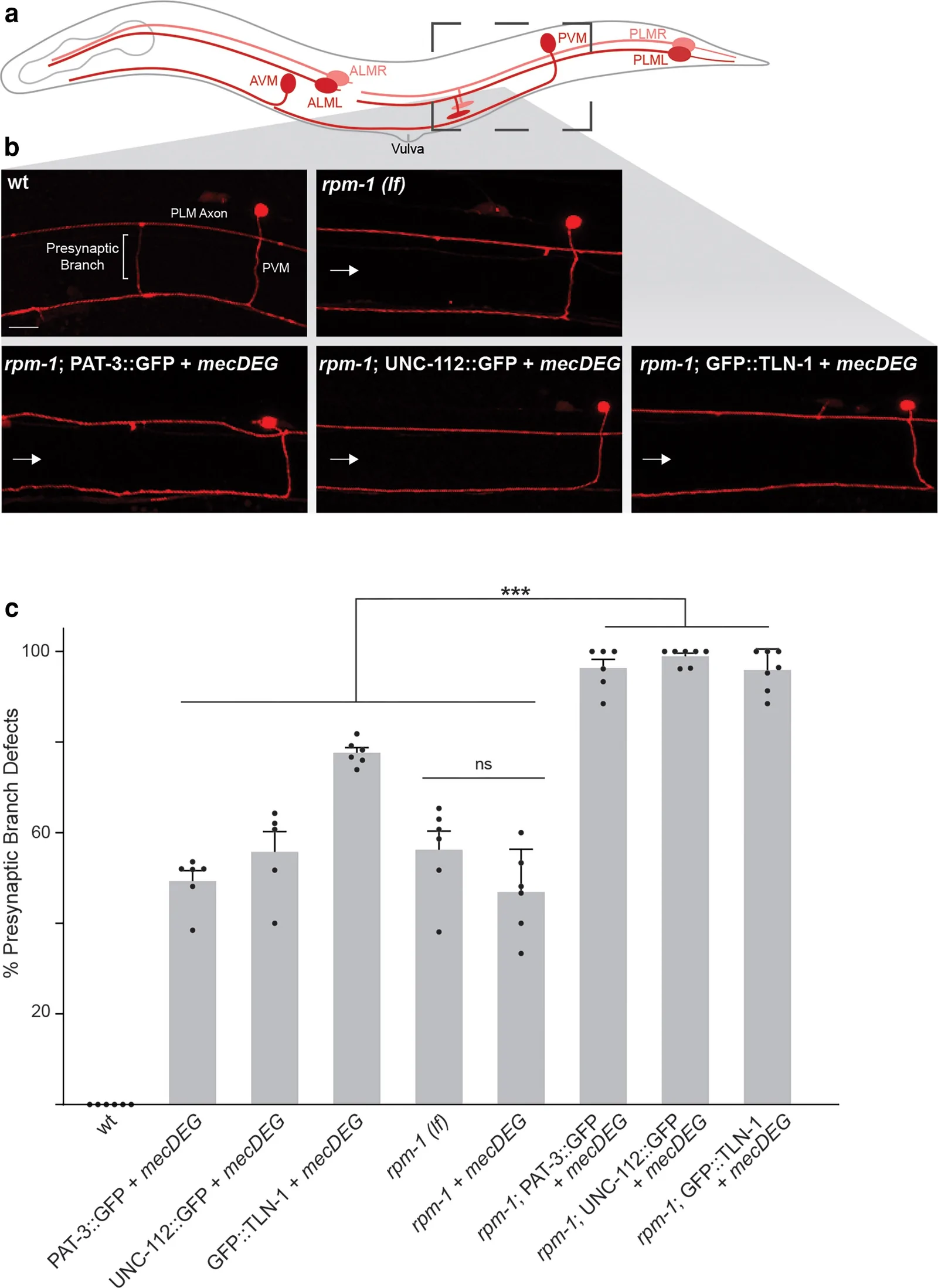
PAT-3/UNC-112/TLN-1 axis interacts genetically with RPM-1 to regulate presynaptic branch development. a) Schematic of mechanosensory neurons highlighting collateral presynaptic branch of PLM neurons (dashed box). P*mec-7*::mRFP (*jsIs973*) labels mechanosensory neurons. b) Representative images of young adults showing PLM branch defects for indicated genotypes. Note branch defects (arrows) occur in *rpm-1 (lf)* mutants, *mecDEG* targeting PAT-3/UNC-112/TLN-1 axis, and *rpm-1* double mutants with PAT-3/UNC-112/TLN-1 axis degradation (e.g. *rpm-1*; PAT-3::GFP + *mecDEG*). c) Quantitation of presynaptic branch defects in PLM neurons for indicated genotypes. Increased frequency of branch defects occurs in *rpm-1* double mutants with PAT-3/UNC-112/TLN-1 axis degradation (e.g. *rpm-1*; PAT-3::GFP+*mecDEG*). Mean (bars) are shown for 5 or more counts (black dots) with 20 or more animals/count for each genotype. Error bars indicate SEM. Significance assessed using two-way ANOVA with Bonferroni correction for multiple comparisons. ***P < 0.001. Scale bar 5 μm.

To examine genetic interactions between RPM-1 and the PAT-3/UNC-112/TLN-1 axis, we combined *mecDEG*-mediated degradation of PAT-3::GFP, UNC-112::GFP or GFP::TLN-1 with an *rpm-1* (lf) mutant. Consistent with earlier experiments (Fig. 2), *mecDEG*-mediated degradation of PAT-3::GFP, UNC-112::GFP or GFP::TLN-1 CRISPR alone resulted in branch defects in PLM neurons similar to *rpm-1* (lf) single mutants (Fig. 4b and c). As an additional control, we tested the *mecDEG* system in *rpm-1* mutants and found that it did not affect the frequency of branch defects associated with *rpm-1* (Fig. 4c). Double mutants for *rpm-1* and components in the PAT-3/UNC-112/TLN-1 axis (e.g. *rpm-1*; PAT-3::GFP+*mecDEG*) exhibited nearly complete absence of branches (Fig. 4b and c). These results suggest *rpm-1* and the TLN-1 axis function in parallel to regulate branch formation. However, we note two caveats. First, while the *rpm-1* mutant we used is a protein null (AlAbdi et al. 2023), *mecDEG* targeting the TLN-1 axis is not a protein null. As noted earlier, *pat-3*, *unc-112*, and likely *tln-1* nulls are not viable and could not be tested. Second, presynaptic branch defects are essentially saturated in double mutants for the TLN-1 axis and *rpm-1*. Thus, we are cautious in stating that RPM-1 functions in parallel to the TLN-1 axis, but this is the most reasonable interpretation of our results.

Next, we wanted to delve into the mechanism behind presynaptic branch defects that occur when the TLN-1 axis is impaired. To do so, we turned to developmental time-course analysis to resolve whether branch defects occur because of defective presynaptic bouton assembly, reduced presynaptic maintenance, or both. Due to the technical complexity of these experiments, we focused on examining *tln-1* single mutants (GFP::TLN-1+ *mecDEG*), *rpm-1* single mutants, and double mutants (*rpm-1;* GFP::TLN-1 + *mecDEG*). We also used only a single control strain (GFP::TLN-1). Consistent with prior time-course studies, we observed presynaptic boutons were fully present by 6 h post-hatch (6 h PH) in wild-type animals (Fig. 5a–d). Similarly, GFP::TLN-1 negative controls fully formed presynaptic branches and boutons by 6 h PH. In both wild-type and control animals, presynaptic boutons were maintained across 16 and 48 h developmental timepoints (Fig. 5b, c, and e). Consistent with prior observations on *rpm-1* mutants (Borgen et al. 2019), presynaptic bouton assembly was delayed at 6 h PH (Fig. 5b–d), but reached similar levels of assembly to wild-type animals by 16 h PH (Fig. 5b, c, and e). However, by 48 h PH presynaptic boutons destabilized resulting in branch retraction (Fig. 5b, c, and f). All of these observations on *rpm-1* mutants reproduce findings from a prior study that showed RPM-1 primarily regulates presynaptic terminal maintenance (Borgen et al. 2019).

**Fig. 5.**
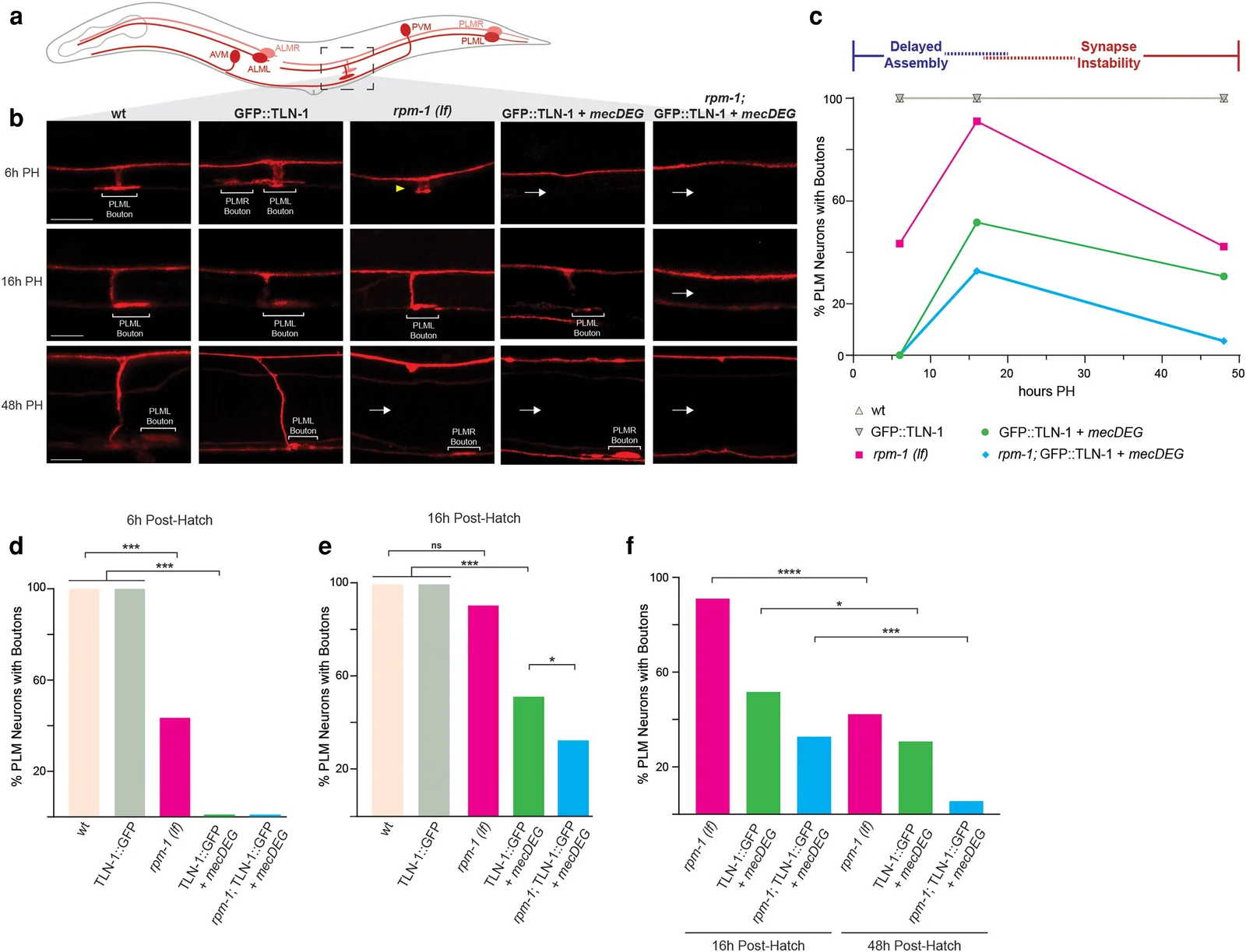
Developmental time-course studies indicate TLN-1 functions in parallel with RPM-1 to regulate presynaptic bouton assembly and maintenance. a) Schematic of mechanosensory neurons highlighting presynaptic branches and boutons of PLM neurons (dashed box). P*mec-7*::mRFP (*jsIs973*) labels mechanosensory neurons. b) Representative images of PLM presynaptic branches and boutons for indicated genotypes are shown for 6, 16 and 48 h post-hatch (PH). Shown are presynaptic PLM boutons (brackets), partial or absent presynaptic branches (arrows), and delayed branch formation (arrowhead). Double mutants (*rpm-1*; GFP::TLN-1+*mecDEG*) have delayed presynaptic bouton formation, which is complete by 6 h PH in wt animals. By 16 h PH, double mutants have reduced presynaptic bouton formation with nearly all boutons destabilized by 48 h PH. c) Summary of quantitative results for presynaptic bouton frequency during development for indicated genotypes. d) Quantitation of presynaptic boutons observed in PLM neurons at 6 h PH for indicated genotypes. e) Quantitation of presynaptic boutons observed in PLM neurons at 16 h PH for indicated genotypes. f) Expanded quantitation for presynaptic boutons observed in PLM neurons at 16 and 48 h PH for indicated genotypes. 16 h was re-plotted to facilitate analysis across time points. Controls for 16 h are shown in c and e), and controls for 48 h are shown in c). For c to f), 50–60 PLM neurons were scored for each time point and genotype. Mean (bars) are shown for each genotype. Significance assessed using Fisher's exact test. ****P < 0.0001, ***P < 0.001, *P < 0.05, ns = not significant. Scale bars 5 μm.

For *tln-1* mutants (GFP::TLN-1 + *mecDEG*), we observed more substantial delays in presynaptic bouton assembly at 6 h PH which did improve by 16 h PH but never reached levels observed in wild-type or GFP::TLN-1 control animals (Fig. 5b–e). Again, similar to *rpm-1* mutants, we observed reduced maintenance of presynaptic bouton maintenance by 48 h PH (Fig. 5b, c, and f). These observations suggest that TLN-1 could be affecting both branch extension and maintenance.

Interestingly, double mutants between *tln-1* and *rpm-1* (*rpm-1*; GFP::TLN-1+*mecDEG*) mirrored a substantial reduction in presynaptic bouton assembly at 6 h PH, similar to *tln-1* single mutants (Fig. 5b–d). The 16 h PH time point was particularly informative, as it revealed a significant reduction in bouton assembly in double mutants compared to *tln-1* single mutants (Fig. 5b, c, and e). Importantly, this time point was not phenotypically saturated, and genetic enhancement suggests TLN-1 and RPM-1 function in parallel to regulate presynaptic bouton development. By 48 h PH, the majority of presynaptic boutons were destabilized in double mutants (*rpm-1*; TLN-1::GFP +*mecDEG*) (Fig. 5b, c, and f).

Together, these findings support several conclusions. (1) TLN-1 plays a primary role in presynaptic branch extension and bouton formation, and a lesser role in bouton maintenance. (2) Impairing TLN-1 or RPM-1 results in similar developmental phenotypes where both delayed presynaptic branch extension and bouton formation occur, and subsequent destabilization of presynaptic boutons occurs. (3) Finally, genetic enhancer effects between *tln-1* and *rpm-1* that occur at 16 h PH (where bouton defects are not saturated) suggest that TLN-1 and RPM-1 function in parallel to regulate presynaptic bouton development in mechanosensory neurons.

### Behavioral habituation to repeated mechanosensory stimulation requires TLN-1

Habituation is a conserved form of simple short-term learning that occurs from *C. elegans* through vertebrates (Glanzman 2010; Bozorgmehr et al. 2013; Randlett et al. 2019; McDiarmid et al. 2020). *C. elegans* rely upon their mechanosensory neurons to sense gentle touch and principally use gap junctions to facilitate the initial touch response (Fig. 6a). Glutamatergic chemical synapses formed by the presynaptic branch mediate habituation to repeated mechanosensory stimulation (Fig. 6a). Similarly, mammalian low-threshold mechanoreceptors communicate via glutamatergic synapses to detect mechanical stimuli (Chalfie et al. 1985; Abraira and Ginty 2013; Chen and Chalfie 2014; Desbois and Grill 2024). Behavioral habituation occurs when an animal's response to repeated mechanical stimulus progressively decreases. Having shown that TLN-1 affects presynaptic branch and bouton development in PLM mechanosensory neurons, we wanted to evaluate whether TLN-1 affects habituation.

**Fig. 6.**
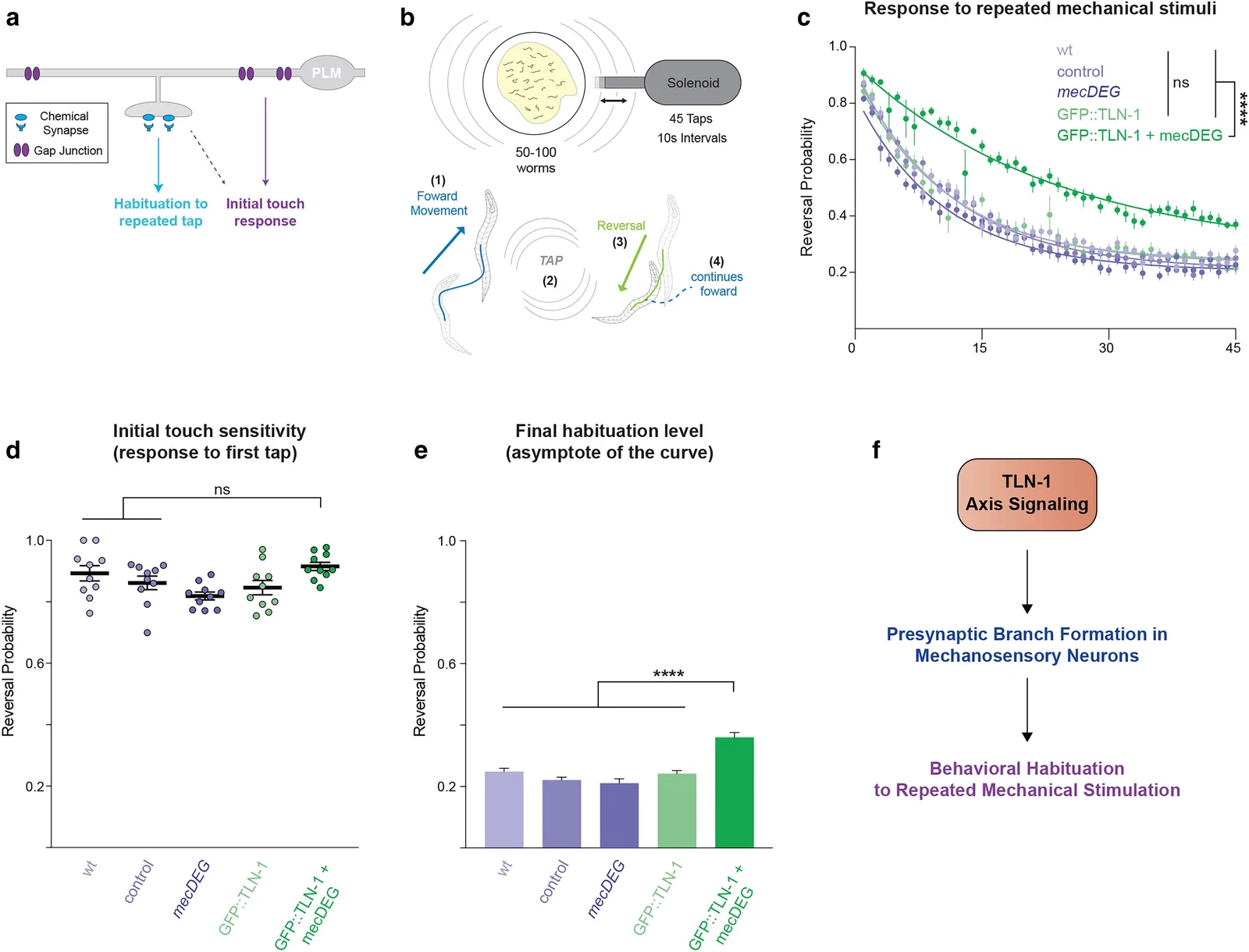
Impairing TLN-1 disrupts behavioral habituation to mechanosensory stimulation. a) PLM mechanosensory neurons mediate habituation through chemical synapses and rely upon electrical synapses for initial touch response. b) Schematic of tap stimulus and habituation protocol where MWT gauges reversal response following tap stimulus. c) Quantitation of response to repeated mechanical stimulus for indicated genotypes. P*mec-7*::mRFP (*jsIs973*, control) labels mechanosensory neurons for branch phenotype verification prior to behavioral testing. Defects in overall habituation response are observed in GFP::TLN-1+*mecDEG* animals compared to controls. d) Quantitation of initial touch response for indicated genotypes based on data from the first tap event. e) Quantitation of final habituation level (asymptote) for indicated genotypes. Defects in final habituation level are observed in GFP::TLN-1+*mecDEG* animals compared to controls. f) Schematic summarizes model for how TLN-1 signaling affects presynaptic branch formation in mechanosensory neurons to influence behavioral habituation to repeated mechanical stimulation. For c, dots represent mean reversal probability for given tap event derived from 10 plates (50–100 animals/plate) acquired over 5 independent biological experiments for each genotype. Error bars are SEM. Significance was determined using two-way ANOVA. For d and e), means (bars) are shown from 10 plates (dots) with 50–100 animals/plate acquired from 5 independent biological experiments for each genotype. Error bars indicate SEM. Significance assessed using two-way ANOVA with Bonferroni correction for multiple comparisons. ****P < 0.0001, ns = not significant.

We employed a Multi-Worm Tracker (MWT) system to computationally deliver and monitor behavioral responses to repeated plate-tap stimuli, a form of low threshold mechanical stimulation (Fig. 6b). MWT simultaneously monitors the locomotor activity of ∼80 individual worms on a plate during each plate tap. Upon tapping, *C. elegans* respond by reversing their direction of movement. MWT assesses reversal probability to each tap event, which is integrated into an overall *C. elegans* population response to repeated mechanical stimulation (Fig. 6c). MWT also allows us to assess the initial tap sensitivity to the first stimulus (Fig. 6d) and derive a final habituation level (asymptote of the curve) for animals tested over a full series of tap events (Fig. 6e).

We began behavioral studies by assessing 4 control strains: wildtype, P*mec-7*::mRFP, *mecDEG*, and GFP::TLN-1 animals. Behavioral experiments were performed using P*mec-7*::RFP to ensure anatomical presynaptic branch phenotypes could be monitored and qualitatively verified prior to behavioral testing. As in prior studies (Giles et al. 2015; Crawley et al. 2017), wild-type animals habituated to repeated tap with decreased responses over time (Fig. 6c and e). Similar levels of initial tap response (Fig. 6d) and habituation to repeated taps also occurred with all control strains (Fig. 6c and e). Quantitative results indicated that the initial tap response, which measures touch sensitivity, was not significantly increased in *tln-1* mutants (GFP::TLN-1+*mecDEG*) compared to wt and controls (Fig. 6d).

Interestingly, habituation was impaired in *tln-1* mutants compared to all controls (Fig. 6c and e). The overall habituation response to repeated mechanical stimulation was significantly impaired in *tln-1* mutants (GFP::TLN-1+*mecDEG*) (Fig. 6c). A second quantitative metric of impaired habituation, the final habituation level, was also significantly increased in *tln-1* mutants compared to all 4 control strains (Fig. 6e).

These results demonstrate that mechanosensory habituation, a simple form of learning in response to mechanical stimulation, is substantially impaired when TLN-1 is degraded specifically in mechanosensory neurons. Taken together with outcomes from anatomical and developmental time-course experiments, our results indicate that TLN-1 functions cell-autonomously in mechanosensory neurons to shape presynaptic branch development thereby influencing mechanosensory-driven behavior (Fig. 6f).

## Discussion

Here, we show that the PAT-3/UNC-112/TLN-1 axis functions cell-autonomously in mechanosensory neurons to regulate presynaptic branch development and habituation to repeated mechanosensory input (Fig. 6f). Our findings further indicate that loss of TLN-1 and activation of TLN-1 to promote inside-out signaling both result in impaired branch formation. Our results suggest that the PAT-3/UNC-112/TLN-1 axis functions in parallel with the RPM-1 ubiquitin ligase signaling hub leading to effects on presynaptic branch development. Finally, we demonstrate that TLN-1 cell-autonomous effects on branch development result in impaired behavioral habituation to mechanical stimulation.

Several of our observations combined with previous findings indicate PAT-3, UNC-112, and TLN-1 form a signaling axis that regulates presynaptic branch development. First, we found that all 3 components of this TLN-1 axis localized to presynaptic branches (Fig. 1). This aligns with prior studies that showed these evolutionarily conserved players form Integrin adhesion complexes in various nonneuronal cell types (Harburger and Calderwood 2009; Zaidel-Bar 2009), and a previous study that indicated the PAT-3/UNC-112/TLN-1 axis regulates axon termination in *C. elegans* (Amezquita et al. 2024). Second, we observed similar defects in branch formation when *mecDEG* was used to cell-autonomously impair PAT-3, UNC-112, or TLN-1 (Fig. 2). Third, independent studies showed that RNAi knockdown for PAT3, UNC-112, and TLN-1 results in altered touch sensitivity (Chen and Chalfie 2014). Interestingly, prior work from our group and others has indicated that the PAT-3/UNC-112/TLN-1 axis is part of a larger signaling network called the adhesome (Zaidel-Bar et al. 2007; Schiller et al. 2011; Winograd-Katz et al. 2014; Amezquita et al. 2024). Our finding that the PAT-3/UNC-112/TLN-1 adhesome axis affects presynaptic branch development raises questions about whether other adhesome players might regulate axon branch development in mechanosensory neurons or more broadly in the nervous system. Importantly, *C. elegans* mechanosensory neurons share developmental and functional similarities with mechanosensory neurons in *Drosophila*, low-threshold mechanoreceptors (LTMRs) in mammals, and Rohon–Beard (Rb) neurons of larval zebrafish (Chalfie 2009; Zimmerman et al. 2014; Desbois and Grill 2024). Thus, it might be valuable to investigate whether the Integrin–Talin axis shapes axon branch development in these other organismal models of mechanosensation.

Our genetic and developmental time-course studies indicate the PAT-3/UNC-112/TLN-1 axis functions in parallel with the RPM-1 signaling hub to regulate presynaptic branch development (Figs. 4 and 5). Interestingly, prior work showed that impairing the PAT-3/UNC-112/TLN-1 axis results in premature axon termination and undergrowth, and genetic interaction studies indicated that the TLN-1 axis displays upstream inhibitory effects on RPM-1 in the context of axon termination in mechanosensory neurons (Amezquita et al. 2024). The most plausible explanation for these findings is that the TLN-1 axis has differing functional relationships with RPM-1 depending upon the subcellular location and cellular process being examined. This interpretation is consistent with earlier studies that showed regulatory proteins, such as the tubulin acetyltransferase ATAT-2, display differing functional relationships in presynaptic branch development and axon termination in mechanosensory neurons (Borgen et al. 2017; Borgen et al. 2019; Teoh et al. 2022). Nonetheless, our results do provide further evidence suggesting that RPM-1 could be a functional component of the *C. elegans* adhesome, which builds upon prior observations (Amezquita et al. 2024).

Our finding that the PAT-3/UNC-112/TLN-1 axis functions in parallel with RPM-1 further highlights shared genetic links to human neurodevelopmental abnormalities. Clinical observations combined with CRISPR engineering in *C. elegans* demonstrated that human genetic variants in RPM-1 (also called MYCBP2) cause a childhood neurodevelopmental disorder that features developmental delay and intellectual disability (Neale et al. 2012; Kosmicki et al. 2017; AlAbdi et al. 2023). Previous large-scale human genetic studies also identified genetic variants in PAT-3 (β-Integrin) and UNC-112 (Kindlin) that are associated with neurodevelopmental abnormalities (Neale et al. 2012; Kosmicki et al. 2017; Amezquita et al. 2024). Thus, our findings on presynaptic branch development tie together multiple genetic players associated with human neurobehavioral deficits.

Interestingly, our findings suggest that balanced TLN-1 signaling is required for branch formation. First, we found TLN-1 loss of function via *mecDEG* targeting results in robust branch defects and impaired presynaptic bouton formation (Figs. 2 and 5). Second, we also show that branch development is regulated by activation of TLN-1 which promotes inside-out Integrin signaling (Fig. 3). The simplest model derived from our results is that a precise balance of Talin inside-out and outside-in signaling is needed for presynaptic branch formation. This supports the previously proposed concept that Talin is a signaling hub that integrates extracellular and intracellular inputs from mechanical stimulation (Goult et al. 2018). Because we used the *mecDEG* as a cell-specific genetic strategy to impair TLN-1, our results suggest balanced TLN-1 signaling is likely to occur cell-autonomously within mechanosensory neurons. However, because TLN-1 activation experiments relied upon a pan-neuronal promoter, we do not rule out the possibility that inside-out signaling in other neurons could influence PLM branch development. Prior studies in lamprey using a peptide-based inhibitor that only affected Talin binding to PIP kinase showed that Talin affects presynaptic actin dynamics and synaptic vesicle accumulation (Morgan et al. 2004). Our findings with an expanded genetic toolkit demonstrate that Talin has a more prominent role in presynaptic branch and bouton formation than previously realized.

At present, very little is known about how Talin affects behavior. Here, we examined how TLN-1 affects habituation, which is an ancient, conserved form of learning (Glanzman 2010; Bozorgmehr et al. 2013; Randlett et al. 2019; McDiarmid et al. 2020). Our findings indicate that TLN-1 functions cell-autonomously in mechanosensory neurons to regulate behavioral habituation to repeated mechanical stimulation (Fig. 6). Interestingly, prior work in flies and mice showed that Talin regulates presynaptic homeostatic plasticity at the neuromuscular junction (Orr et al. 2022). Our results indicate that an Integrin–Talin axis and Talin inside-out signaling regulate presynaptic branch development in a different neuronal context to shape behavioral plasticity. Interestingly, observations from cell-based studies outside the nervous system have been used to propose that Talin mediates mechanosensory memory, which has been termed the MeshCODE theory (Goult 2021; Goult et al. 2021). Our organismal results support this postulate by demonstrating that Talin is required for mechanosensory presynaptic branch development and learning. Whether the Integrin/Kindlin/Talin axis regulates learning and memory in response to mechanical stimulation in other organismal models and behavioral paradigms awaits further investigation.

## Supplementary Material

jkag154_Supplementary_Data

## Data Availability

Strains and plasmids are available upon request. The authors affirm that all data necessary for confirming the conclusions of the article are present within the article, figures, tables and Supplementary materials. Metadata and statistical analysis (prism) for all quantitative experiments can be found with the following DOI (10.5061/dryad.msbcc2g8w) on the Dryad website (https://datadryad.org): https://doi.org/10.5061/dryad.msbcc2g8w. Supplemental material available at G3 online.

## References

[jkag154-B1] Abraira VE, Ginty DD. 2013. The sensory neurons of touch. Neuron. 79:618–639. 10.1016/j.neuron.2013.07.051. PMC3811145 23972592

[jkag154-B2] AlAbdi L et al 2023. Loss-of-function variants in MYCBP2 cause neurobehavioural phenotypes and corpus callosum defects. Brain. 146:1373–1387. 10.1093/brain/awac364. PMC10319777 36200388

[jkag154-B3] Amezquita J et al 2024. Integrin adhesome axis inhibits the RPM-1 ubiquitin ligase signaling hub to regulate growth cone and axon development. PLoS Genet. 20:e1011496. 10.1371/journal.pgen.1011496. PMC11642917 39671436

[jkag154-B4] Arnaout MA, Mahalingam B, Xiong JP. 2005. Integrin structure, allostery, and bidirectional signaling. Annu Rev Cell Dev Biol. 21:381–410. 10.1146/annurev.cellbio.21.090704.151217. 16212500

[jkag154-B5] Borgen MA, Giles AC, Wang D, Grill B. 2019. Synapse maintenance is impacted by ATAT-2 tubulin acetyltransferase activity and the RPM-1 signaling hub. Elife. 8:e44040. 10.7554/eLife.44040. PMC6355192 30652969

[jkag154-B6] Borgen MA, Wang D, Grill B. 2017. RPM-1 regulates axon termination by affecting growth cone collapse and microtubule stability. Development. 144:4658–4672. 10.1242/dev.154187. PMC5769622 29084805

[jkag154-B7] Bozorgmehr T, Ardiel EL, McEwan AH, Rankin CH. 2013. Mechanisms of plasticity in a *Caenorhabditis elegans* mechanosensory circuit. Front Physiol. 4:88. 10.3389/fphys.2013.00088. PMC3750945 23986713

[jkag154-B8] Calderwood DA et al 1999. The Talin head domain binds to integrin beta subunit cytoplasmic tails and regulates integrin activation. J Biol Chem. 274:28071–28074. 10.1074/jbc.274.40.28071. 10497155

[jkag154-B9] Calderwood DA, Campbell ID, Critchley DR. 2013. Talins and kindlins: partners in integrin-mediated adhesion. Nat Rev Mol Cell Biol. 14:503–517. 10.1038/nrm3624. PMC4116690 23860236

[jkag154-B10] Chalfie M et al 1985. The neural circuit for touch sensitivity in *Caenorhabditis elegans*. J Neurosci. 5:956–964. 10.1523/JNEUROSCI.05-04-00956.1985. PMC6565008 3981252

[jkag154-B11] Chalfie M . 2009. Neurosensory mechanotransduction. Nat Rev Mol Cell Biol. 10:44–52. 10.1038/nrm2595. 19197331

[jkag154-B12] Chavis P, Westbrook G. 2001. Integrins mediate functional pre- and postsynaptic maturation at a hippocampal synapse. Nature. 411:317–321. 10.1038/35077101. 11357135

[jkag154-B13] Chen X, Chalfie M. 2014. Modulation of *C. elegans* touch sensitivity is integrated at multiple levels. J Neurosci. 34:6522–6536. 10.1523/JNEUROSCI.0022-14.2014. PMC4012311 24806678

[jkag154-B14] Cingolani LA, Goda Y. 2008. Differential involvement of beta3 integrin in pre- and postsynaptic forms of adaptation to chronic activity deprivation. Neuron Glia Biol. 4:179–187. 10.1017/S1740925X0999024X. 19758485

[jkag154-B15] Clegg DO, Wingerd KL, Hikita ST, Tolhurst EC. 2003. Integrins in the development, function and dysfunction of the nervous system. Front Biosci. 8:d723–d750. 10.2741/1020. 12700040

[jkag154-B16] Cram EJ, Clark SG, Schwarzbauer JE. 2003. Talin loss-of-function uncovers roles in cell contractility and migration in *C. elegans*. J Cell Sci. 116:3871–3878. 10.1242/jcs.00705. 12915588

[jkag154-B17] Crawley O et al 2017. A MIG-15/JNK-1 MAP kinase cascade opposes RPM-1 signaling in synapse formation and learning. PLoS Genet. 13:e1007095. 10.1371/journal.pgen.1007095. PMC5754208 29228003

[jkag154-B18] Cui Y, Rolova T, Fagerholm SC. 2024. The role of integrins in brain health and neurodegenerative diseases. Eur J Cell Biol. 103:151441. 10.1016/j.ejcb.2024.151441. 39002282

[jkag154-B19] Desbois M, Grill B. 2024. Molecular regulation of axon termination in mechanosensory neurons. Development. 151:dev202945. 10.1242/dev.202945. PMC11698068 39268828

[jkag154-B20] Eysert F et al 2021. Correction to: Alzheimer's genetic risk factor FERMT2 (Kindlin-2) controls axonal growth and synaptic plasticity in an APP-dependent manner. Mol Psychiatry. 26:5608. 10.1038/s41380-020-01015-8. PMC8758472 33398090

[jkag154-B21] Giles AC, Opperman KJ, Rankin CH, Grill B. 2015. Developmental function of the PHR protein RPM-1 is required for learning in *Caenorhabditis elegans*. G3 (Bethesda). 5:2745–2757. 10.1534/g3.115.021410. PMC4683646 26464359

[jkag154-B22] Glanzman DL . 2010. Common mechanisms of synaptic plasticity in vertebrates and invertebrates. Curr Biol. 20:R31–R36. 10.1016/j.cub.2009.10.023. PMC5125775 20152143

[jkag154-B23] Goult BT et al 2009. The structure of an interdomain complex that regulates talin activity. J Biol Chem. 284:15097–15106. 10.1074/jbc.M900078200. PMC2685691 19297334

[jkag154-B24] Goult BT . 2021. The mechanical basis of memory—the MeshCODE theory. Front Mol Neurosci. 14:592951. 10.3389/fnmol.2021.592951. PMC7947202 33716664

[jkag154-B25] Goult BT, Brown NH, Schwartz MA. 2021. Talin in mechanotransduction and mechanomemory at a glance. J Cell Sci. 134:jcs258749. 10.1242/jcs.258749. PMC8697387 34708856

[jkag154-B26] Goult BT, Yan J, Schwartz MA. 2018. Talin as a mechanosensitive signaling hub. J Cell Biol. 217:3776–3784. 10.1083/jcb.201808061. PMC6219721 30254032

[jkag154-B27] Haage A et al 2018. Talin autoinhibition regulates cell-ECM adhesion dynamics and wound healing in vivo. Cell Rep. 25:2401–2416.e5. 10.1016/j.celrep.2018.10.098. 30485809

[jkag154-B28] Harburger DS, Calderwood DA. 2009. Integrin signalling at a glance. J Cell Sci. 122:159–163. 10.1242/jcs.018093. PMC2714413 19118207

[jkag154-B29] Huang Z et al 2006. Distinct roles of the beta 1-class integrins at the developing and the mature hippocampal excitatory synapse. J Neurosci. 26:11208–11219. 10.1523/JNEUROSCI.3526-06.2006. PMC2693048 17065460

[jkag154-B30] Hynes RO . 2002. Integrins: bidirectional, allosteric signaling machines. Cell. 110:673–687. 10.1016/S0092-8674(02)00971-6. 12297042

[jkag154-B31] Jaudon F, Thalhammer A, Cingolani LA. 2021. Integrin adhesion in brain assembly: from molecular structure to neuropsychiatric disorders. Eur J Neurosci. 53:3831–3850. 10.1111/ejn.14859. 32531845

[jkag154-B32] Kerstein PC, Patel KM, Gomez TM. 2017. Calpain-mediated proteolysis of Talin and FAK regulates adhesion dynamics necessary for axon guidance. J Neurosci. 37:1568–1580. 10.1523/JNEUROSCI.2769-16.2016. PMC5299572 28069919

[jkag154-B33] Kim M, Carman CV, Springer TA. 2003. Bidirectional transmembrane signaling by cytoplasmic domain separation in integrins. Science. 301:1720–1725. 10.1126/science.1084174. 14500982

[jkag154-B34] Klapholz B, Brown NH. 2017. Talin—the master of integrin adhesions. J Cell Sci. 130:2435–2446. 10.1242/jcs.190991. 28701514

[jkag154-B35] Kosmicki JA et al 2017. Refining the role of de novo protein-truncating variants in neurodevelopmental disorders by using population reference samples. Nat Genet. 49:504–510. 10.1038/ng.3789. PMC5496244 28191890

[jkag154-B36] Lilja J, Ivaska J. 2018. Integrin activity in neuronal connectivity. J Cell Sci. 131:jcs212803. 10.1242/jcs.212803. 29907643

[jkag154-B37] Lu F et al 2022. Mechanism of integrin activation by talin and its cooperation with kindlin. Nat Commun. 13:2362. 10.1038/s41467-022-30117-w. PMC9054839 35488005

[jkag154-B38] McDiarmid TA et al 2020. Systematic phenomics analysis of autism-associated genes reveals parallel networks underlying reversible impairments in habituation. Proc Natl Acad Sci U S A. 117:656–667. 10.1073/pnas.1912049116. PMC6968627 31754030

[jkag154-B39] Montanez E et al 2008. Kindlin-2 controls bidirectional signaling of integrins. Genes Dev. 22:1325–1330. 10.1101/gad.469408. PMC2377186 18483218

[jkag154-B40] Morgan JR et al 2004. A role for talin in presynaptic function. J Cell Biol. 167:43–50. 10.1083/jcb.200406020. PMC2172527 15479735

[jkag154-B41] Nayal A, Webb DJ, Horwitz AF. 2004. Talin: an emerging focal point of adhesion dynamics. Curr Opin Cell Biol. 16:94–98. 10.1016/j.ceb.2003.11.007. 15037311

[jkag154-B42] Neale BM et al 2012. Patterns and rates of exonic de novo mutations in autism spectrum disorders. Nature. 485:242–245. 10.1038/nature11011. PMC3613847 22495311

[jkag154-B43] Orr BO, Fetter RD, Davis GW. 2022. Activation and expansion of presynaptic signaling foci drives presynaptic homeostatic plasticity. Neuron. 110:3743–3759.e3746. 10.1016/j.neuron.2022.08.016. PMC9671843 36087584

[jkag154-B44] Park YK, Goda Y. 2016. Integrins in synapse regulation. Nat Rev Neurosci. 17:745–756. 10.1038/nrn.2016.138. 27811927

[jkag154-B45] Pasterkamp RJ, Peschon JJ, Spriggs MK, Kolodkin AL. 2003. Semaphorin 7A promotes axon outgrowth through integrins and MAPKs. Nature. 424:398–405. 10.1038/nature01790. 12879062

[jkag154-B46] Poinat P et al 2002. A conserved interaction between beta1 integrin/PAT-3 and Nck-interacting kinase/MIG-15 that mediates commissural axon navigation in *C. elegans*. Curr Biol. 12:622–631. 10.1016/S0960-9822(02)00764-9. 11967148

[jkag154-B47] Randlett O et al 2019. Distributed plasticity drives visual habituation learning in larval zebrafish. Curr Biol. 29:1337–1345.e4. 10.1016/j.cub.2019.02.039. PMC6545104 30955936

[jkag154-B48] Rogalski TM, Mullen GP, Gilbert MM, Williams BD, Moerman DG. 2000. The UNC-112 gene in *Caenorhabditis elegans* encodes a novel component of cell-matrix adhesion structures required for integrin localization in the muscle cell membrane. J Cell Biol. 150:253–264. 10.1083/jcb.150.1.253. PMC2185566 10893272

[jkag154-B49] Schaefer AM, Hadwiger GD, Nonet ML. 2000. rpm-1, a conserved neuronal gene that regulates targeting and synaptogenesis in *C. elegans*. Neuron. 26:345–356. 10.1016/S0896-6273(00)81168-X. 10839354

[jkag154-B50] Schiller HB, Friedel CC, Boulegue C, Fässler R. 2011. Quantitative proteomics of the integrin adhesome show a myosin II-dependent recruitment of LIM domain proteins. EMBO Rep. 12:259–266. 10.1038/embor.2011.5. PMC3059911 21311561

[jkag154-B51] Sepers JJ, Ramalho JJ, Kroll JR, Schmidt R, Boxem M. 2022. ERM-1 phosphorylation and NRFL-1 redundantly control lumen formation in the *C. elegans* intestine. Front Cell Dev Biol. 10:769862. 10.3389/fcell.2022.769862. PMC8860247 35198555

[jkag154-B52] Sydor AM, Su AL, Wang FS, Xu A, Jay DG. 1996. Talin and vinculin play distinct roles in filopodial motility in the neuronal growth cone. J Cell Biol. 134:1197–1207. 10.1083/jcb.134.5.1197. PMC2120982 8794861

[jkag154-B53] Tan CL et al 2012. Kindlin-1 enhances axon growth on inhibitory chondroitin sulfate proteoglycans and promotes sensory axon regeneration. J Neurosci. 32:7325–7335. 10.1523/JNEUROSCI.5472-11.2012. PMC6622300 22623678

[jkag154-B54] Tan CL et al 2015. Full length talin stimulates integrin activation and axon regeneration. Mol Cell Neurosci. 68:1–8. 10.1016/j.mcn.2015.03.011. PMC4604251 25771432

[jkag154-B55] Teoh JS et al 2022. Synaptic branch stability is mediated by non-enzymatic functions of MEC-17/alphaTAT1 and ATAT-2. Sci Rep. 12:14003. 10.1038/s41598-022-18333-2. PMC9385713 35977998

[jkag154-B56] Walser M, Umbricht CA, Frohli E, Nanni P, Hajnal A. 2017. β-integrin de-phosphorylation by the density-enhanced phosphatase DEP-1 attenuates EGFR signaling in *C. elegans*. PLoS Genet. 13:e1006592. 10.1371/journal.pgen.1006592. PMC5305270 28135265

[jkag154-B57] Wang S et al 2017. A toolkit for GFP-mediated tissue-specific protein degradation in *C. elegans*. Development. 144:2694–2701. 10.1242/dev.150094. PMC5536931 28619826

[jkag154-B58] Warren MS et al 2012. Integrin beta1 signals through Arg to regulate postnatal dendritic arborization, synapse density, and behavior. J Neurosci. 32:2824–2834. 10.1523/JNEUROSCI.3942-11.2012. PMC3313657 22357865

[jkag154-B59] Williams BD, Waterston RH. 1994. Genes critical for muscle development and function in *Caenorhabditis elegans* identified through lethal mutations. J Cell Biol. 124:475–490. 10.1083/jcb.124.4.475. PMC2119919 8106547

[jkag154-B60] Winograd-Katz SE, Fässler R, Geiger B, Legate KR. 2014. The integrin adhesome: from genes and proteins to human disease. Nat Rev Mol Cell Biol. 15:273–288. 10.1038/nrm3769. 24651544

[jkag154-B61] Zaidel-Bar R . 2009. Evolution of complexity in the integrin adhesome. J Cell Biol. 186:317–321. 10.1083/jcb.200811067. PMC2728394 19667126

[jkag154-B62] Zaidel-Bar R, Itzkovitz S, Ma'ayan A, Iyengar R, Geiger B. 2007. Functional atlas of the integrin adhesome. Nat Cell Biol. 9:858–867. 10.1038/ncb0807-858. PMC2735470 17671451

[jkag154-B63] Zimmerman A, Bai L, Ginty DD. 2014. The gentle touch receptors of mammalian skin. Science. 346:950–954. https://www.science.org/doi/10.1126/science.1254229 10.1126/science.1254229 PMC4450345 25414303

