# Supplementary material for: Integrin–Talin axis regulates mechanosensory presynaptic development and behavioral habituation to mechanical stimulation
Source: G3 (Bethesda). 2026 Jun 16;16(10):jkag154. doi: 10.1093/g3journal/jkag154 (PMC13643791; doi:10.1093/g3journal/jkag154)
Supplement: Supplementary file 1 [file jkag154_supplementary_data.docx]

**Integrin-Talin axis regulates mechanosensory presynaptic development and behavioral habituation to mechanical stimulation**

Jonathan Amezquita^1,2 *^, Nelson A. Ayala^1,3 *^, Muriel Desbois^4^, Neal D. Mathew^1,5^, Karla J. Opperman^1^, Brock Grill^1,3,5^

^1^ Norcliffe Foundation Center for Integrative Brain Research, Seattle Children's Research Institute, Seattle, 98101, USA.

^2^ Molecular and Cellular Biology Graduate Program, University of Washington, Seattle, 98101 USA.

^3^ Department of Pharmacology, University of Washington School of Medicine, Seattle, 98101 USA.

^4^ School of Life Sciences, Keele University, Keele, ST5 5BG, UK.

^5^ Department of Pediatrics, University of Washington School of Medicine, Seattle, 98101 USA.

^*^ Both authors contributed equally

Correspondence: brock.grill@seattlechildrens.org

**Supplementary Figures and Tables**

**Figure S1 and S2**

**Table S1-S4**

**Figure S1:** **Expanded dataset showing components of PAT-3/UNC-112/TLN-1 axis are localized to presynaptic branches of PLM mechanosensory neurons. a)** Schematic of *C. elegans* mechanosensory neurons highlighting collateral presynaptic branches of PLM neurons visualized using P*mec-7*::mRFP (*jsIs973*) (dashed box). **b-d)** Additional images showing CRISPR engineered PAT-3::GFP, UNC-112::GFP and GFP::TLN-1 are localized to collateral branches (bracket). Images show only a single PLM branch on one side of the animal. Note PAT-3/UNC-112/TLN-1 axis components also show prominent expression in primary axons of PLM neurons and body wall muscles. Scale bar 5μm.

**Figure S2: Expanded dataset showing *mecDEG* reduces GFP::TLN-1 fluorescence levels in PLM neuron soma and proximal axon. a-d)** Further representative examples of Airyscan super-resolution images of young adults showing GFP::TLN-1 is reduced in PLM neurons where *mecDEG* is expressed compared to controls (GFP::TLN-1 alone). Regions of interest (ROI) used for quantitative analysis in Figure 2 h-k are shown for PLM soma (yellow, dashed oval) and proximal axon (yellow box). Note far right panels show GFP::TLN-1 fluorescence intensity as heatmap. Scale bar 2μm.

**Table S1: Transgenic and CRISPR Strains**

| **Figure** | **Strain** | **Genotype** |
| --- | --- | --- |
| Figure 1, 2 | XMN1242 | *pat-3*(*bgg86* [PAT-3::GFP CRISPR]); *jsIs973* [P_mec-7_mRFP; unc-119 (+)] III |
| Figure 1, 2 | XMN1168 | *jsIs973* [P_mec-7_mRFP, unc-119(+)] III; *unc-112*(*bgg68* [UNC-112::GFP CRISPR]) V |
| Figure 1, 2, 5, 7 | XMN1231 | *tln-1*(*zh117* [GFP::TLN-1 CRISPR]) I; *jsIs973* [P_mec-7_mRFP, unc-119 (+)] III |
| Figure 2, 3, 4, 5, 6, 7 | XMN913 | *jsIs973* [P_mec-7_mRFP, unc-119(+)] III |
| Figure 2, 7 | XMN1167 | *itSi953* [P_mec-18_*mecDEG*, unc-119(+)] II; *jsIs973* [P_mec-7_mRFP, unc-119(+)] III |
| Figure 2, 4 | XMN1243 | *itSi953* [P_mec-18_*mecDEG*, unc-119(+)] II; *pat-3*(*bgg86* [PAT-3::GFP]) *jsIs973* [P_mec-7_mRFP, unc-119(+)] III |
| Figure 2, 4 | XMN1359 | *itSi953* [P_mec-18_*mecDEG*, unc-119(+)] II; *jsIs973* [P_mec-7_mRFP, unc-119(+)] III; *unc-112*(*bgg68* [UNC-112::GFP CRISPR]) V |
| Figure 2, 4, 5, 6, 7 | XMN1230 | *tln-1*(*zh117* [GFP::TLN-1 CRISPR]) I; *itSi953* [P_mec-18_*mecDEG*, unc-119(+)] II; *jsIs973* [P_mec-7_mRFP, unc-119(+)] III |
| Figure 2 | XMN1229 | *tln-1*(*ok1648*) I; *jsIs973* [P_mec-7_mRFP, unc-119(+)] III |
| Figure 2 | XMN1412 | *tln-1*(*zh117* [GFP::TLN-1 CRISPR]) I; *itSi953* [P_mec-18_*mecDEG*, unc-119(+)] II; *jsIs973* [P_mec-7_mRFP, unc-119(+)] III; *bggEx172* (P_rgef-1_FLAG::TLN-1, P_rps-27_NeoR) |
| Figure 4, 5, 6 | XMN1152 | *jsIs973* [P_mec-7_mRFP, unc-119(+)] III; *rpm-1(ju44)* V |
| Figure 4 | XMN1272 | *itSi953* [P_mec-18_*mecDEG*, unc-119(+)] II; *jsIs973* [P_mec-7_mRFP, unc-119(+)] III; *rpm-1(ju44)* V |
| Figure 4 | XMN1366 | *itSi953* [P_mec-18_*mecDEG*, unc-119(+)] II; *pat-3*(*bgg86* [PAT-3::GFP]) *jsIs973* [P_mec-7_mRFP, unc-119(+)] III; *rpm-1(ju44)* V |
| Figure 4 | XMN1258 | *itSi953* [P_mec-18_*mecDEG*, unc-119(+)] II; *jsIs973* [P_mec-7_mRFP, unc-119(+)] III; *rpm-1 (ju44)*; *unc-112*(*bgg68* [UNC-112::GFP]) V |
| Figure 4, 5 | XMN1305 | *rpm-1(ju44)* V; *tln-1*(*zh117* [GFP::TLN-1 CRISPR] I;  *itSi953* [P_mec-18_*mecDEG*, unc-119(+)] II; *jsIs973* [P_mec-7_mRFP, unc-119(+)] III |

*mecDEG =* ltSi953 [mec-18p::vhhGFP4::zif-1::operon-linker::mKate::tbb-2 3'UTR + Cbr-unc-119(+)] II

**Table S2: Primers for genotyping**

| **Gene** | **Allele/ Construct** | **Primer Sequence** |
| --- | --- | --- |
| *mecDEG* Transgene | *itSi953* | ttTi5605 fwd: 5’ TTTCTCAGTTGTGATACGGTTTTT 3’  Int: 5’ aggaacagaataacagatgatgagc 3’  ttTi5605 rev: 5’ CGCTACTTACCGGAAACCAA 3’ |
| *tln-1* | *ok1648* | ok1648 fwd: 5’ gagccaaatgacgagtaggg 3’  wt rev: 5’ AGAGTCGAACGGATGTTTCG 3’  ok1648 rev: 5’ tctcatggatcgctttttcg 3’ |
| *tln-1* | *zh117* | zh117 fwd: 5’ TCCAGATAAACCGCAAATCC 3’  zh117 Int: 5’ GGCAGACAAACAAAAGAATGG 3’  zh117 rev: 5’ ATCTGGGCGTGGTTTGTTAG 3’ |
| *pat-3* | *bgg86* | bgg86 fwd: 5’ TTATGGCCAAATGGGATACG 3’  bgg86 rev: 5’ TGTGAGTTGTTGGTCGGTGT 3’  bgg86 Int: 5’ GGCAGACAAACAAAAGAATGG 3’ |
| *unc-112* | *bgg68* | wt fwd: 5’ CGAAGCAAAGAACATTCTCAA 3’  bgg68 fwd: 5’ GGCAGACAAACAAAAGAATGG 3’  wt rev: 5’ TTGTACCTACGTTTGCCTAC 3’ |
| *rpm-1* | *ju44* | *ju44* fwd: 5’ CGTGTATGACCTGTAAACGAGAAG 3’  *ju44* rev: 5’ GACATGTTGGAAGAAGATGTTTTG 3’  Digest with AccI |

**Table S3: Injection Conditions**

| **Figure** | **Transgene** | **Injected Strain** | **Injection Mix** |
| --- | --- | --- | --- |
| **Figures 1, 2, 4** | PAT-3::GFP CRISPR (*bgg86*) | N2 | 4uL Cas9 |
|  |  |  | 5.0uL IDT tracrRNA (0.8ug/uL) |
|  |  |  | 2.8uL IDT crRNA (0.8ug/uL) |
|  |  |  | 1.6uL pRF4 (rol-6 plasmid) |
|  |  |  | 2ug hybrid repair template |
| **Figures 1, 2, 4** | UNC-112::GFP CRISPR (*bgg68*) | N2 | 5μL tracrRNA (4μg/μL) |
|  |  |  | 0.4μL dpy-10 crRNA (8μg/μL) |
|  |  |  | 0.55μL dpy-10 repair ssODN (500μg/μL) |
|  |  |  | 1μL UNC-112::GFP crRNA (8ug/μL) |
|  |  |  | 6.8μL UNC-112::GFP PCR Template (500ng/μL) |
|  |  |  | 0.5μL KCl (1M) |
|  |  |  | 0.75uL Hepes pH 7.4 (200mM) |
|  |  |  | 4.6μL Nuclease-Free H_2_0 |
|  |  |  | 5μL Cas9 (10μg/μL) |
| **Figure 2** | TLN-1 Rescue  (*bggEx172*) | *tln-1*(*zh117* [GFP::TLN-1]) I; *itSi953* [P_mec-18_*mecDEG*, *unc-119*(+)] II; *jsIs973* [P_mec-_*_7_*mRFP, *unc-119*(+)] III | 65ng/μL pBluescript (pBG-49) |
|  |  |  | 10ng/μL P_rps-27_NeoR (pBG-264) |
|  |  |  | 25ng/μL P_rgef-1_FLAG::TLN-1 (pBG-GY1100) |
| **Figure 3** | OE  TLN-1  (2 lines) | *jsIs973* [P_mec-_*_7_*mRFP, *unc-119*(+)] III | 50ng/uL P_ttx-3_RFP |
|  |  |  | 100ng/uL P_rgef-1_FLAG::TLN-1 WT |
| **Figure 3** | OE  TLN-1 *T1767E*  (3 lines) | *jsIs973* [P_mec-_*_7_*mRFP, *unc-119*(+)] III | 50ng/uL P_ttx-3_RFP |
|  |  |  | 100ng/uL P_rgef-1_FLAG::TLN-1 *T1767E* |
| **Figure 3** | OE  TLN-1 *E1770A*  (2 lines) | *jsIs973* [P_mec-_*_7_*mRFP, *unc-119*(+)] III | 50ng/uL P_ttx-3_RFP |
|  |  |  | 100ng/uL P_rgef-1_FLAG::TLN-1 *E1770A* |

**Table S4: CRISPR Targeting Sequences and Repair Templates**

| **Gene** | **crRNA** | **crRNA Target Sequence** | **Repair Template** |
| --- | --- | --- | --- |
| *unc-112* | UNC-112::GFP | CTTTTCCACAAACTTACAGG | GCAAAGAACATTCTCAAAATCTTGATGAAGAACTTTTCCATAAGCTCACTGG***AGG***ATGG GCTGGAGGAGGAGGATCCGGAGGAGGAGGATCCGGAGGAGGAGGATCC --- GFP ---  TAGATATTTAAATTTCTATAATCTTTTGCAAACCA |
| *pat-3* | PAT-3::GFP | AAGGATAAAAACTATTTAGT | PCR products containing 120bp of homology arms and no homology arms were mixed, melted, and annealed to create a hybrid dsDNA repair template with 120bp single-stranded overhang (Dokshin et al, Genetics, 2018)  No gene homology repair template  GGAGGAGGAGGATCTGGTGGTGGAGGATCTGGTGGAGGTGGATCA ---GFP----  Long homology repair template  aagtgacacggaacaaaaaacatataaatttatcaaattatcatttttcagAACGAGAACCCAATCTACAAACAGGCCACGACAACATTTAAAAATCCAGTATACGCTGGAAAAG***CCA***ACGGAGGAGGAGGATCTGGTGGTGGAGGATCTGGTGGAGGTGGATCA ---GFP----atagtttttatccttatattttaataattttcccaaattttctaatatgaaagctcaatttctccatccaaacaactcgaaacgagtatttagcgataaattgtcacattcttctttgttttattcaaaaaatct |

Legend: underline (crRNA targeting sequence), Italic bold (Pam sequence), Red (Insertion), Blue (silent mutations in repair to prevent Cas9 re-cutting), Orange (linker)
